# Botulinum toxin-induced masseter muscle atrophy is associated with impaired autophagic flux without signs of apoptosis in mice

**DOI:** 10.1038/s41420-026-02982-7

**Published:** 2026-02-28

**Authors:** Esteban R. Quezada, Noelia Blanco, Paola Llanos, Alfredo Criollo, Mario Chiong, Sonja Buvinic

**Affiliations:** 1 Escuela de Enfermería, Facultad de Salud, Universidad Santo Tomás, Santiago, Chile; 2 Institute for Research in Dental Sciences, Faculty of Dentistry, Universidad de Chile, Independencia, Chile; 3 Center for Exercise, Metabolism and Cancer, CEMC-2016, Faculty of Medicine, Universidad de Chile, Independencia, Chile; 4 Advanced Center for Chronic Diseases (ACCDiS), Faculty of Chemical and Pharmaceutical Sciences, Universidad de Chile, Independencia, Chile

**Keywords:** Physiology, Apoptosis, Autophagy, Macroautophagy, Preclinical research

## Abstract

Botulinum toxin type A (BoNTA) injection into the masseter muscle is widely used for clinical and esthetic purposes. Masseter muscle atrophy is a secondary effect of transient neuromuscular blockade induced by BoNTA. While muscle atrophy has been linked to enhanced ubiquitin-proteasome system activity, leading to increased protein degradation, the role of other catabolic pathways, such as apoptosis and autophagy, remains understudied. In the present study, we evaluated these cellular processes in a mice model of unilateral injection of BoNTA in the masseter muscle, and its relationship with muscle atrophy. Changes in neither molecular markers of apoptosis (cleaved caspase-3, cleaved PARP) nor DNA fragmentation were observed in BoNTA-injected muscles. Conversely, a significant accumulation of the autophagy markers microtubule-associated proteins 1 A/1B light chain 3B (LC3), sequestosome 1 (SQSTM1/p62), and BCL2-associated athanogene 3 (BAG3), along with a reduction in muscle fiber diameter, was observed at 7 days post-BoNTA. These changes were not affected by autophagic flux blockade with chloroquine. Interestingly, LC3 accumulation positively correlates with masseter mass reduction induced by BoNTA. These findings suggest that BoNTA disrupts skeletal muscle homeostasis, promoting atrophy through impaired autophagic activity. Our results not only shed light on the mechanism of BoNTA-induced muscle atrophy but also has broader implications for understanding and potentially treating a range of muscle wasting disorders.

## Introduction

Temporomandibular disorders (TMD) manifest as pain and functional impairment in the jaw joint and associated muscles [1]. Among the primary muscles involved in mastication, the masseter muscle plays a significant role due to its powerful force generation [2]. Hyperactivity and hypertrophy of the masseter muscle contribute to TMD development [2, 3]. One of the therapeutic options for neuromuscular-origin TMD is the injection of Botulinum Toxin Type A (BoNTA) into the masseter and/or temporal muscle, leading to paralysis, and volume reduction of muscles [4]. However, in addition to its reversible paralysis effects, studies in both preclinical models and patients have shown that BoNTA injections lead to sustained muscle atrophy [5, 6]. Beyond therapeutic applications, BoNTA injections into the masseter muscle also have esthetic purposes, for a slimmer facial appearance, conforming to social requirements. [2]. Nevertheless, owing to the lack of studies verifying its effectiveness and safety, the use of BoNTA in the masseter muscle is not approved by either the marketing companies or the U.S. Food and Drug Administration (FDA) [7]. This *off-label* use highlights the need for comprehensive research in this field.

Muscle atrophy is a condition characterized by a reduction in size and alteration of tissue structure [8]. It is associated with a decrease in the cross-sectional area of muscle fibers and the protein synthesis/degradation ratio, leading to weakened strength [8–10]. It has been proposed that muscle atrophy is driven by a complex cellular mechanism, which may include the activation of the ubiquitin-proteasome pathway, the induction of apoptosis, and/or the regulation of autophagy. The ubiquitin-proteasome pathway induces the expression of the ubiquitin-E3 ligases Atrogin-1/MAFbx and MuRF1 through a transcription factor FoxO-dependent mechanism, promoting protein degradation [11]. In rats with tibialis anterior atrophy evoked by surgical denervation, increases in TUNEL-positive nuclei and abundance of Bax protein were observed, along with decreased levels of Bcl-2 protein and muscle mitochondrial oxygen consumption, suggesting the occurrence of apoptosis [12]. It has been proposed that this mechanism triggers muscle atrophy by reducing protein synthesis due to a loss in the number of nuclei per muscle fiber [13]. Furthermore, 14 days after mice sciatic nerve denervation, a significant increase in LC3-II was described in tibialis anterior, suggesting concurrent activation of autophagy [14]. In mice, unilateral injection of BoNTA in the masseter muscle increases the expression of muscle atrophy markers Atrogin-1/MAFbx and MuRF1 from 2 days post-injection [15], along with a reduction in the cross-sectional area of muscle fibers from 7 days post-injection [16]. However, no information is available on the induction of apoptosis or autophagy in the masseter muscle during atrophy evoked by BoNTA.

In this work, we aimed to evaluate the induction of apoptosis and autophagy in the masseter muscle of adult mice evokedby BoNTA injection and its relationship with the consequent muscle atrophy.

## Results

### BoNTA injection into the masseter muscle does not induce apoptosis

**Fig. 1:**
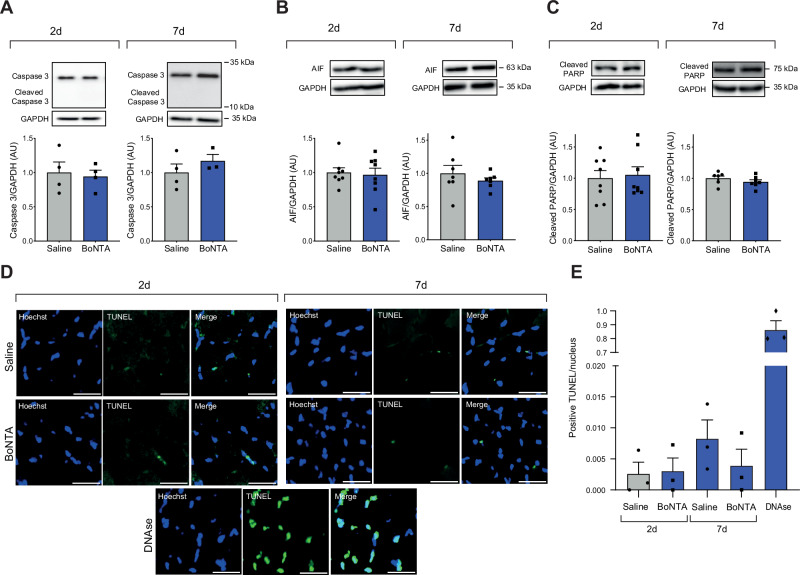
BoNTA injection does not induce apoptosis in the masseter muscle. Contralateral masseter muscles were injected with 0.2 U of BoNTA or saline. Proteins were extracted from dissected muscles 2 or 7 days after intervention. No changes were observed in the relative levels of caspase-3, cleaved caspase-3 (**A**), AIF (**B**), and cleaved PARP (**C**) at 2 and 7 days. GAPDH was used as a loading control. Values are presented as mean ± SEM (*n* = 3–8). A representative image is shown above each graph. No significant differences were found in saline vs BoNTA, Mann–Whitney test. Transverse cryosections were made, and TUNEL (green) and Hoechst (blue) staining were evaluated using 20x epifluorescence microscopy. BoNTA did not increase the number of TUNEL-positive nuclei at 2 and 7 days post injection (**D**). DNase was used as a positive control for DNA fragmentation. In all images, the white scale bar equals 50 µm. Data are presented as the ratio of the number of TUNEL-positive nuclei per total nuclei (**E**). Values are presented as mean ± SEM (*n* = 3). No significant differences were found in saline vs BoNTA; Kruskal-Wallis with Dunn’s post hoc.

To evaluate if BoNTA induces apoptosis in the masseter muscle in vivo, we performed an unilateral injection of BoNTA into C57BL/6 adult male mice, and apoptosis markers (cleaved caspase-3, AIF, cleaved PARP, and TUNEL) were evaluated at 2 and 7 days post-intervention. No variation in total caspase-3 and cleaved caspase-3 levels was observed at 2 and 7 days post-BoNTA (Fig. 1A). Osmotic stress was induced as a positive control for the activation of caspase-3 (Figure S1). Caspase-3 independent apoptosis was assessed by measuring relative levels of AIF. BoNTA did not increase AIF protein levels in the masseter muscle (Fig. 1B). Additionally, no significant changes in cleaved PARP were observed at 2 and 7 days post-BoNTA, compared to saline-injected contralateral muscles (Fig. 1C). In cross-sections of the masseter muscle, less than 1% TUNEL-positive cells were observed post-BoNTA, contrasting with more than 80% TUNEL-positive cells observed with the DNA-fragmentation inducer DNase (Fig. 1D and E). Taken together, these data suggest that BoNTA did not induce apoptosis in the masseter muscle of adult mice within the 2- to 7-day post-injection period.

### BoNTA impairs autophagy flux

**Fig. 2:**
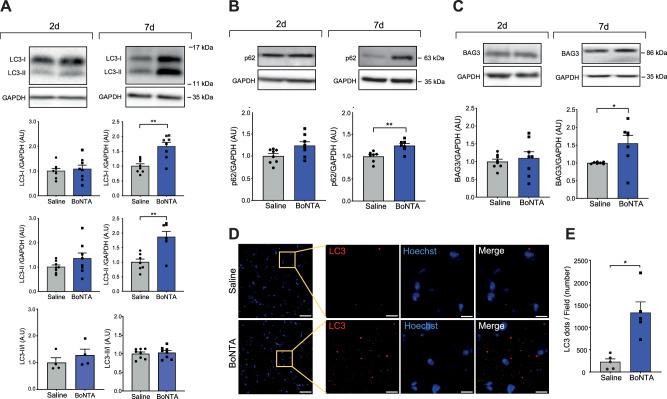
BoNTA increases autophagic markers in the masseter muscle. Contralateral masseter muscles were injected with 0.2 U of BoNTA or saline. Proteins were extracted from muscles dissected 2 or 7 days after intervention and analyzed by western blot. GAPDH was used as a loading control. No changes in LC3-I or LC3-II levels were observed at 2 days post-injection, but BoNTA increased the relative levels at 7 days, without changing the LC3II/LC3I ratio (**A**). No changes in SQSTM1/p62 were observed at 2 days, but relative SQSTM1/p62 levels increased at 7 days post BoNTA (**B**). No changes were observed in BAG3 at 2 days post-BoNTA but a significant increase was detected at 7 days (**C**). Representative images of Western blots are shown. Values are presented as mean ± SEM. (*n* = 6–8). *p < 0.05, **p < 0.01; Mann-Whitney test. Contralateral masseter muscles were dissected and frozen 7 days after injection. Transverse cryosections were obtained, and LC3 (red) and nuclei staining (blue) were evaluated. Representative images acquired with a 20x confocal microscope are shown. In each case, a wide-field image is displayed, followed by the enlarged image of the field outlined by the yellow box (**D**). The number of autophagic vesicles (LC3/red) was quantified in the entire field (**E**) (*n* = 4-5). *p < 0.05; Mann-Whitney test.

LC3-I, LC3-II, SQSTM1/p62, and BAG3 protein relative levels were evaluated to assess autophagy. A significant increase in LC3-I and LC3-II was observed at 7 days but not at 2 days post-BoNTA (Fig. 2A), as well as an increase in LC3 mRNA levels (Figure S2). Similarly, an increase in LC3-I and LC3-II was observed at 14 days post-BoNTA (Figure S3). In addition, a significant increase in both SQSTM1/p62 and BAG3 protein levels was observed from 7 days post-BoNTA (Fig. 2B, C). In transverse cryosections of the mouse masseter muscle, total LC3 labeling was evaluated by indirect immunofluorescence. The number of dots per field increased 7 days after injection with BoNTA, suggesting the formation of autophagosomes (Fig. 2D, E).

The increase in autophagy markers in the mouse masseter muscle after injection with BoNTA could be explained by activation of autophagy or by blocking the final stages of autophagy. To differentiate between these two options, we quantified SQSTM1/p62 accumulation and colocalization with LC3. Subsequently, chloroquine (CQ) was administered intraperitoneally (IP) to block autophagic flux and determine whether the changes induced by BoNTA corresponded to an activation or inhibition of the process.

**Fig. 3:**
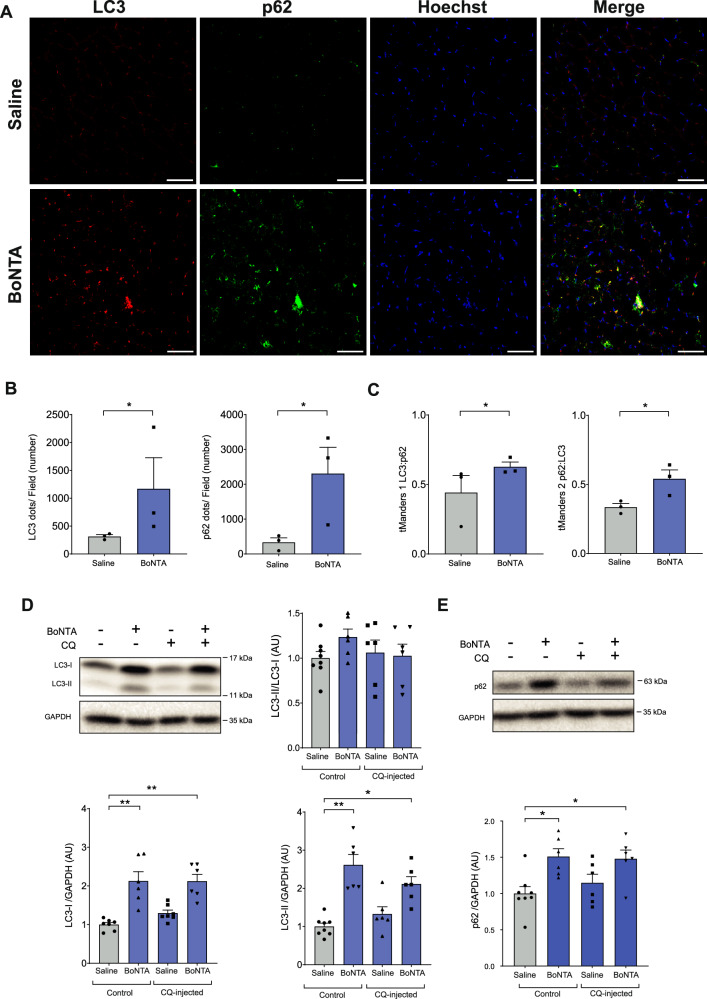
BoNTA induces blockage of autophagic flux in the masseter muscle. **A** The contralateral masseter muscles were injected with 0.2 U of BoNTA or saline solution. Transverse cryosection slides were obtained, and LC3 (red), SQSTM1/p62 (green), and nuclei staining (blue) were evaluated. Representative images acquired with a 40X confocal microscope are shown; scale bar: 50 µm. BoNTA increased the relative levels of dots per field (**B**) and colocalization (**C**) of LC3/red and SQSTM1/p62/green (*n* = 3). *p < 0.05; Mann-Whitney test. **D**, **E** Contralateral masseter muscles were injected with 0.2 U of BoNTA, or saline solution, in the absence or presence of IP injection of chloroquine (CQ) 15 mg/kg. Proteins were extracted from the muscles 7 days later, separated by SDS-PAGE, and analyzed by Western blot. The combination of BoNTA and CQ did not further increase the accumulation of the autophagy-related proteins LC3 (**D**) or SQSTM1/p62 (**E**) compared with BoNTA alone. GAPDH was used as a loading control. Values are represented as mean ± SEM (*n* = 6–8). *p < 0.05; **p < 0.01; Kruskal-Wallis test with Dunn’s post hoc.

In transverse cryosections of the mice masseter muscle, the number of LC3 and SQSTM1/p62 dots per field (Fig. 3A, B) and their colocalization (Fig. 3C) increased 7 days after injection with BoNTA. A dose-response experiment was performed to determine the appropriate CQ dose (15–50–100 mg/kg) by evaluating the accumulation of LC3-I, LC3-II, SQSTM1/p62, and the LC3-II/LC3-I ratio in the masseter muscle. A significant increase in the LC3-I and SQSTM1/p62 was observed with 15 mg/kg CQ, but not with 50 mg/kg CQ (Figure S4), while 100 mg/kg was lethal. Furthermore, we used a dry-fasting model in mice to verify whether 15 mg/kg CQ effectively blocks autophagic flux [17]; 50 mg/kg was not used because it was ineffective. A significantly increased LC3-II/LC3-I ratio was observed when comparing fasting + CQ with fasting alone (Figure S5). On the other hand, to rule out the previously described cytotoxic effects of CQ [17], we evaluated DNA fragmentation. TUNEL-positive nuclei were not increased in the masseter muscle of mice treated with 15 mg/kg CQ (Figure S6).

BoNTA injection into the masseter muscle evoked a significant increase in the relative levels of LC3-I, LC3-II, and SQSTM1/p62, both in control mice and in 15 mg/kg CQ-injected mice. No difference in those protein levels was observed between BoNTA and BoNTA+CQ conditions (Fig. 3D, E). All these results strongly suggest that injection of BoNTA into the masseter muscle blocks autophagic flux.

### Masseter muscle atrophy induced by BoNTA injection is not dependent on autophagy activation

**Fig. 4:**
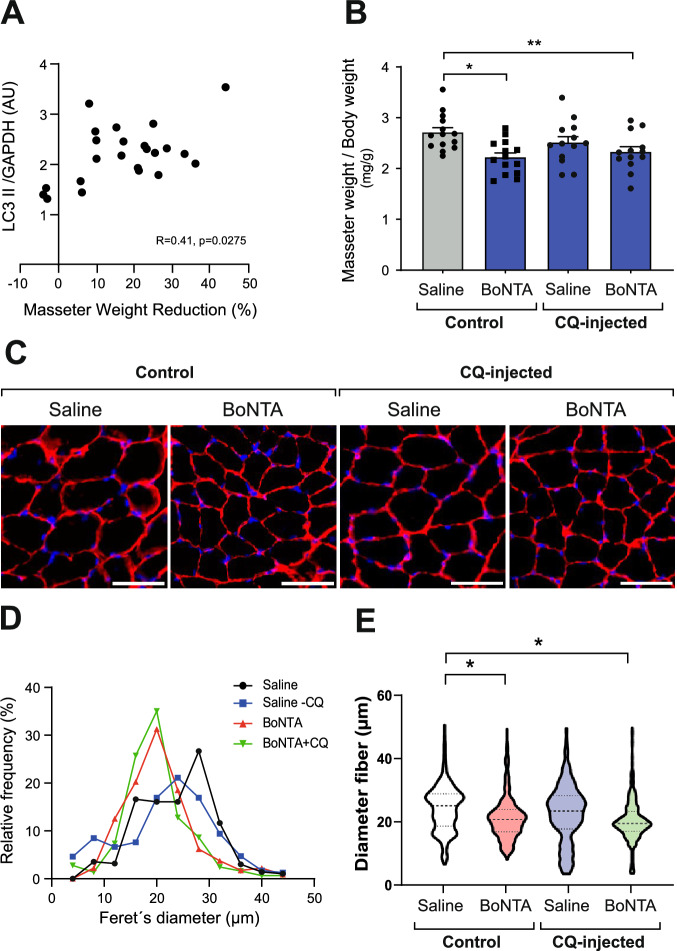
BoNTA-induced masseter muscle atrophy is not related to the blockage of autophagic flux. Contralateral masseter muscles were injected with 0.2 U of BoNTA, or saline, in the absence or presence of IP injection of chloroquine (CQ) 15 mg/kg. Proteins were extracted from dissected muscles 7 days after the intervention. There was a correlation between LC3-lI levels and masseter muscle mass reduction after BoNTA administration (**A**). Spearman’s correlation was used. (*n* = 23). *p* < 0.05. Masseter muscle mass from all experimental groups was assessed at 7 days (**B**). Values are represented as mean ± SEM. (*n* = 13-14). *p < 0.05; **p < 0.01. A one-way ANOVA was used, followed by Dunnett’s multiple comparisons test. Transverse cryosections and immunofluorescence were performed to detect cell limit (caveolin 3, red) and nuclei (Hoechst, blue) (**C**). Scale bar: 50 µm. Histogram of minimum Feret diameter for each of the conditions (**D**). (*n* = 5) *, *p* < 0.05. Nested t-test (**E**). The histogram (**D**) and nested t-test (**E**) were obtained from 192 -575 fibers per masseter per animal.

To explore the relationship between autophagy and muscle atrophy, we evaluated structural and morphological changes in the masseter muscle following BoNTA injection by measuring muscle mass and muscle fiber diameter. We observed a significant positive-correlation between LC3-II levels and the reduction in masseter muscle mass following BoNTA administration (Fig. 4A). Additionally, as previously described by our group [16], BoNTA injection significantly reduced the masseter mass, and the minimum Feret diameter of muscle fibers compared to the contralateral saline-injected masseter (Fig. 4B–D).

**Fig. 5:**
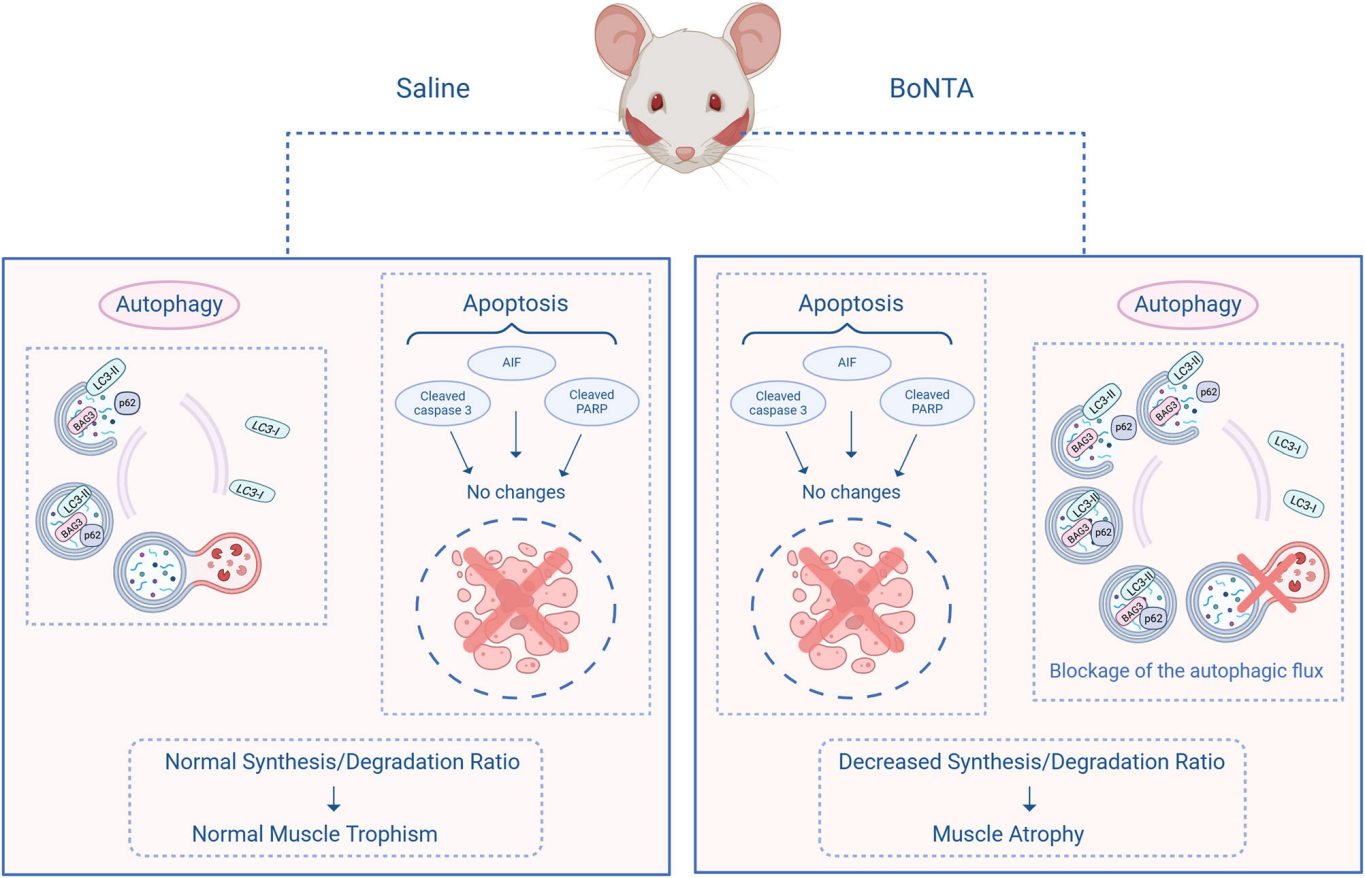
Model of the role of apoptosis and autophagy in BoNTA-induced mouse masseter muscle atrophy. The unilateral injection of BoNTA results in flaccid paralysis and consequent atrophy of the mouse masseter muscle. BoNTA does not induce apoptosis in the masseter muscle at 2 or 7 days post-injection. However, increases in autophagy markers were observed at 7 days post-BoNTA. Because chloroquine did not change the levels of these autophagy markers, the results suggest that autophagic flux is blocked, likely due to impaired autophagosome–lysosome fusion. The blockade of autophagic flux does not directly affect protein degradation during BoNTA-induced muscle atrophy. Created in BioRender. Buvinic, S. (2026) https://BioRender.com/e87g834.

The autophagy blockade with CQ did not significantly decrease the minimum Feret diameter compared to the control, as depicted in the violin plot (Fig. 4E). Only a tendency toward a reduction in diameter was observed, visualized as a shift to the left in the histogram of the Feret diameter quantification (Fig. 4D). In contrast, BoNTA + CQ reduces masseter muscle fiber diameter as well as BoNTA (Fig. 4D, E). In summary, although BoNTA induces autophagy markers, and these are directly associated with muscle loss, blocking autophagic flux with CQ does not prevent BoNTA-induced atrophy. This reinforces the idea that BoNTA does not activate but rather blocks autophagy in the masseter muscle Fig. 5.

## Discussion

This work aimed to evaluate the induction of apoptosis and autophagy in the masseter muscle of adult mice evoked by BoNTA injection and its relationship with the consequent muscle atrophy. We demonstrated that BoNTA did not induce apoptosis but suggests a blocked the autophagy flux in the masseter muscle. LC3-II accumulation, a marker of autophagic flux blockade, is positively associated with BoNTA-induced muscle wasting. To our knowledge, this is the first study to report the effects of BoNTA on autophagy in skeletal muscle, which may complement and relate to its previously described effects on proteasomal protein degradation [16] in the regulation of muscle loss.

Building on earlier findings [16], BoNTA induces masseter atrophy by day 7, with further progression by day 14, while atrophy-related gene expression begins as early as day 2 [15]. Therefore, we selected 2 and 7 days post-BoNTA to assess apoptosis and autophagy, both before morphological changes become apparent and once the atrophic phenotype is clearly established.

Our data showed that the cleaved caspase-3, AIF, and cleaved PARP levels did not change upon BoNTA injection. Therefore, it is suggested that BoNTA treatment did not activate apoptosis in the masseter muscle. Furthermore, no changes were observed in TUNEL-positive nuclei. This also discards other cell death mechanisms that involve DNA fragmentation, such as necroptosis, pyroptosis, ferroptosis, and paraptosis [18–21]. This data contrasts with other studies, which have reported apoptosis and positive TUNEL staining in the masseter muscle following BoNTA in bilateral injection models [22, 23]. However, bilateral masseter hypofunction can confound the results by mixing the actual effects of BoNTA with stress in mice due to impaired chewing and reduced feeding. Some research proposes a relationship between apoptosis and a decrease in the number of nuclei in muscle fibers related to atrophy [24]. Nevertheless, the latter is controversial. When the extensor digitorum longus muscle is overloaded due to partial removal of its agonist muscle, tibialis anterior, an increase in the number of nuclei is observed in the hypertrophied fibers. Conversely, when muscle atrophy is induced by denervation in this model, no reduction in the number of nuclei per fiber is observed [25]. All these data suggest that apoptosis is not involved in muscle atrophy.

A few studies have evaluated the relationship of BoNTA with the autophagy process. Bilateral BoNTA injection in the masseter muscle increases LC3-positive cells in the mandibular condyle cartilage of mice [26]. In contrast, BoNTA injection in mice gastrocnemius muscle upregulates mammalian target of rapamycin (mTOR)/S6 kinase signaling pathway, HDAC4, Myog, Fbxo32/MAFbx/Atrogin-1 pathway, and transcription of synaptic components, but not autophagy [27]. BoNTA treatment significantly increases LC3-II/LC3-I and Beclin-1 protein levels in human dermal microvascular endothelial cells [28], and LC3-II in human skin keloid fibroblasts [29]. However, in these studies, the analysis of BoNTA’s effect on autophagy is limited by the absence of autophagic flux blockade [30].

Our data are the first description of the effects of BoNTA on autophagic flux in skeletal muscle. We found that BoNTA-injection increased the relative protein levels of LC3-I, LC3-II, SQSTM1/p62, and BAG3, which suggests an increase in the autophagic flux. However, the accumulation of the protein markers might also be due to a blockage of autophagic flux, which would impair their proper delivery to lysosomes for degradation. This could explain why the increase in both LC3-I and LC3-II does not alter the LC3-II/LC3-I ratio after BoNTA treatment. Activation of autophagic flux typically promotes the lipidation of LC3-I to LC3-II, thereby increasing the LC3-II/LC3-I ratio [30]. However, the absence of such a change in our results, despite the elevated LC3 mRNA levels, suggests enhanced LC3 transcription leading to an accumulation of LC3-I protein, which in turn prevents a shift in the LC3-II/LC3-I ratio. The increased levels and colocalization of SQSTM1/p62 with LC3 suggest an accumulation of autophagosomes [31]. Moreover, the absence of an additional rise in LC3-II following CQ treatment in combination to BoNTA indicates that autophagosome accumulation may have reached a peak [32]. Therefore, we suggest that the effect of BoNTA on the masseter muscle is indeed due to a blockade of autophagic flux. This outcome was unexpected, as we initially hypothesized that activation of this pathway would contribute to the protein degradation underlying muscle atrophy [14, 33].

Our results contradict those described in skeletal muscle atrophy induced by denervation. Surgical denervation of the tibialis anterior muscle shows a dynamic change in autophagy [14]. After three days of denervation, autophagy was slightly reduced in the tibialis anterior muscle. However, autophagic flux increases 7, 14, and 28 days after denervation [14]. In the BoNTA-treated masseter muscle, changes in autophagy markers were not observed on day 2 but increased on day 7, indicating that modification requires long-term exposure to the stimulus. On the other hand, surgical dissection of the sciatic nerve generates a condition of paralysis, added to a pattern of local neuropathy [34]. The neuropathy could add a confounding variable that could explain the differences found in both models of muscle atrophy.

Our data showed that CQ treatment triggers a non-significant decrease in masseter muscle fiber diameter compared to control mice, suggesting that the autophagy flux blockade is insufficient to induce masseter muscle atrophy. This contrasts with previous reports in conditional Atg7 KO mice, where genetic ablation of autophagy promotes muscle atrophy, weakness, and myofiber degeneration [33]. However, the autophagy blockade through a KO intervention is probably more robust than a pharmacological intervention. Masseter muscles derived from mice treated with BoNTA or BoNTA+CQ showed a similar reduction in fiber diameter compared to saline-injected muscles. This outcome was expected, as both interventions block autophagy flux, and their combined atrophic effect has likely reached a maximum. Therefore, our results argue against a role for autophagy induction in BoNTA-induced masseter muscle atrophy. Nonetheless, we cannot exclude the possibility that autophagy inhibition contributes to muscle loss. Future studies using autophagy inducers will be required to determine whether stimulation of this pathway can prevent or reverse BoNTA-induced muscle wasting. For the moment, in the current work, it was observed that LC3-II accumulation—a marker of impaired autophagic flux—was positively associated with BoNTA-induced muscle wasting, further supporting a link between disrupted autophagy and masseter atrophy.

Previous studies have shown that BoNTA injection into the mice masseter muscle increases Atrogin/MAFbx and MuRF-1 RNAm levels, suggesting the activation of the ubiquitin/proteasome pathway [15]. Several studies have revealed the connection between autophagy and ubiquitin/proteasome pathways in different cell types. Blocking autophagy can lead to an upregulation of the ubiquitin-proteasome pathway. In colon cancer cells, knocking down ATG genes using siRNA increased proteasomal subunits’ expression [35]. Conversely, knocking down ATG7 and ATG12 in HeLa-Cells inhibited autophagy and proteasomal activity despite the accumulation of ubiquitinated proteins and SQSTM1/p62 via Nrf1-dependent pathways [36]. In skeletal muscles, the transcription factor FoxO3 enhances the expression of numerous autophagy-related genes and lysosomal proteolysis targets, as well as the ubiquitin-E3 ligases Atrogin-1/MAFbx and MURF1 [37–40]. Inhibition of autophagy leads to muscle atrophy with myopathy phenotypes, which are similar to the effects observed when Atrogin-1 and MuRF1 are disrupted or when there are deficiencies in genes involved in various catabolic pathways [11, 41]. Notably, inhibiting autophagy in Atg7 KO muscles increased the protein levels of Atrogin-1 and MuRF1, along with polyubiquitinated targets. Deletion of Atg7 also induces apoptosis in muscle cells [33]. Muscle-specific Atg5^–/–^ mice exhibit identical phenotypes as Atg7^–/–^ mice [42]. Furthermore, in the C2C12 skeletal muscle cell line, inhibition of the proteasome with MG132 increased SQSTM1/p62 protein levels [43]. These data support the hypothesis that the BoNTA-dependent blockade of autophagic flux could potentiate the activation of the ubiquitin-proteasome pathway and facilitate masseter muscle atrophy. However, further experiments are required to demonstrate this relationship.

Autophagy is associated with muscle tropism and the degradation and recycling of proteins and organelles [44]. Blockade of the autophagic flux has been associated with the accumulation of misfolded proteins and damaged organelles, which can promote myopathies [45]. The inhibition of autophagic flux, induced by muscle-specific BAG3 or ATG7 KO, led to muscle atrophy and myopathy characterized by a decrease in cross-sectional skeletal muscle fiber area, loss of the sarcomere z-line, accumulation of membrane bodies, vacuolization, and mitochondrial swelling [46, 47]. This could suggest that BoNTA-evoked blockade of the autophagic flux would also decrease the muscle fiber’s ability to maintain intracellular homeostasis.

This is the first study to suggest that BoNTA blocks autophagic flux in skeletal muscle and that the accumulation of autophagy markers is positively associated with muscle wasting evoked by BoNTA.

Is relevant to note that the data obtained in this work show the muscular response to a single injection of BoNTA in young male mice. Repeated or prolonged exposure to BoNTA, as occurs in clinical and esthetic practice, could further compromise the function and integrity of skeletal muscles and related tissues. Future studies will be required to determine whether the cellular pathways underlying BoNTA-induced muscle wasting are sexually dimorphic or age-dependent.

## Materials and Methods

### Animals

Male Balb/C mice (8 weeks old, 15–18 gr) were obtained from the Experimental Platform of the Faculty of Dentistry (Universidad de Chile). Mice were maintained in standard room conditions (48-50% humidity; 20 ± 2 °C; 12 h light/dark cycle), with water and food (LabDiet® JL Rat and Mouse/Auto 6 F 5K67; LabDiet, St. Louis, MO, USA) *ad libitum*. Intraperitoneal overdose of anesthesia was used for euthanizing the animals. The proposal has been approved by the IACUC of the Universidad de Chile (N° 21446–ODO–UCH-e2).

### Unilateral injection of BoNTA into the masseter muscle

The animals were subjected to sedation/anesthesia by intraperitoneal injection with a combination of Ketamine/Xylazine (80 mg/Kg and 8 mg/Kg, respectively). Then, each animal received a single injection of BoNTA (0.2 U/10 µL; Onabotulinumtoxin A; BOTOX ®, Allergan North Chicago, IL, USA) in the right masseter (experimental side). The left masseter muscle received the same volume of vehicle (NaCl 0.9% w/v, 10 µL; saline side). The masseter muscles were dissected and processed for biochemical (total extract) or histological (transverse cryosections) analyses at 2 and 7 days post-BoNTA.

### Blockade of autophagy in vivo

The role of autophagy was determined by chloroquine’s pharmacological blockade of autophagic flux (CQ, Sigma-Aldrich, C6628, MO, USA). Four intraperitoneal (IP) injections of 15, 50, or 100 mg/kg CQ were performed for 7 days (−1d, 1 d, 3 d, 6 d). The first dose was injected into the masseter muscle one day before the BoNTA/Saline injection protocol. The experimental groups were Saline, Saline+CQ, BoNTA, and BoNTA+CQ.

### Western Blot

Masseter muscles were processed with a high-energy benchtop homogenizer (BeadBug, Benchmark Scientific, USA) in RIPA lysis buffer (Thermo Fisher Scientific, 89900, IL, USA) supplemented with protease inhibitors (1:200; Calbiochem Set III protease inhibitor cocktail, Fisher Scientific, 539134; Darmstadt, Germany) and phosphatase inhibitors (1:100; phosphatase inhibitor cocktail I, Sigma-Aldrich, P2850; MO, USA). Protein quantification was performed by spectrophotometry at 562 nm using BCA (Pierce™ BCA Protein Assay Kits, Thermo Fisher, 23225, IL, USA). Lysate proteins were resuspended in 1X loading buffer (4X Laemmli protein sample buffer, Bio-Rad, 1610747, CA, USA). Proteins were resolved by electrophoresis on 10-15% polyacrylamide gels under denaturing conditions (SDS-PAGE). Between 20-40 µg of proteins were loaded in each lane. Membranes were blocked at room temperature (RT) for one hour in TBS buffer (50 mM Tris, 150 mM NaCl, pH 7.6) supplemented with 0.1% Tween-20 (Sigma-Aldrich, P1379, USA) (TBS-T) and 5% skim milk. Primary antibodies were incubated at 4°C overnight. Chemiluminescent label visualization was performed using the RapidStepTM ECL Reagent Kit (EDM Millipores, DW100720-500, MA, USA). For proteins with low detection levels, the WESTAR Supernova detection kit (Cyanogen, XLS3, Bologna, Italy) was used, allowing visualization of femtogram levels. The following antibodies were used: caspase-3, AIF, cleaved PARP, LC3, SQSTM1/p62, BAG3, and GAPDH (Table S1). Images were obtained by chemiluminescence on the Amersham imager 600® (GE Healthcare Life Sciences, IL, USA) and analyzed by densitometry with the free access program ImageJ (Version 1.53k).

The full-length, uncropped original western blots used in this manuscript are included in Figure S7.

### Immunofluorescence

Muscles were quickly dissected, preserved in OCT, and frozen for 45–60 sec in isopentane (Sigma-Aldrich, M32631, MO, USA), cooled with liquid nitrogen. Cryosections of 12 μm were made in a cryostat (Leica, CM1520, Nussloch, Germany). Samples were immediately fixed in 4% paraformaldehyde (Electron Microscopy Science, 15700, PA, USA) in 1X PBS for 20 min. Finally, the sections were incubated with the primary antibody overnight at 4°C in a humid chamber. The primary antibodies were LC3 and Caveolin-3 (Table S1). Nuclei were labeled with Hoechst reagent (1:10,000, Invitrogen, 33342, ONT, Canada). Samples were then mounted with Dako anti-fading reagent (Dako, S3023, CA, USA) and stored at 4°C until visualization. LC3 and SQSTM1/p62 images were acquired with a Carl Zeiss Axiovert 135 M-LSM Microsystems confocal microscope (CEMC, Faculty of Medicine, Universidad de Chile) and processed using the ImageJ program (Version 1.53k). Caveolin-3 images were acquired with an inverted epifluorescence microscope (Ti-E, Nikon®, USA) using the 20X objective, and the fiber diameter was quantified using Feter’s minimum diameter with the Dragonfly software (Version 2022.2.0.1409, Object Research Systems, Canada).

### TUNEL

The TUNEL Kit (DeadEnd™ Fluorometric TUNEL System, Promega, G3250, WI, USA) was used on the samples mounted on the slide. 100 μL of equilibration buffer was added for 10 min (RT). The rTdT incubation kit was prepared (45 µL equilibrium buffer + 5 µL mix nucleotides + 1 µL rTdT enzyme), and 50 µL was added per section and incubated in a humid chamber protected from light at 37°C for 60 min. At the end of the incubation, the samples were washed with 2X SSC buffer for 15 min. Nuclei were labeled with Hoechst reagent (1:10,000, Invitrogen, 33342, ONT, Canada) for 5 min (RT). Samples were mounted with SlowFade anti-fading reactive (Invitrogen, S36939) and stored at 4 °C until use. The DNAase kit was a positive control (Promega, M6101, WI, USA). Images were acquired with an inverted epifluorescence microscope (Ti-E, Nikon®, USA) using the 20X objective and processed using ImageJ software (Version 1.53k).

### Statistical analysis

The results were expressed as mean ± standard error of the mean (SEM). The two-tailed paired t-test was used to determine the difference between data groups. The one-tailed one-way ANOVA was used to compare more than two groups, followed by Dunnett’s multiple comparisons tests. In cases where the data did not pass the Shapiro-Wilk normality test, the non-parametric Mann-Whitney and Kruskal-Wallis tests with Dunn’s post hoc were applied. In instances of fiber diameter, with multiple data coming from each individual sample, the two-tailed nested t-test was used for comparisons. The level of significance established was p < 0.05. All statistical analyses were performed in GraphPad Prism 7 (CA, USA).

## Supplementary information

Supplemental material

## Data Availability

The data supporting this study’s findings are available from the corresponding authors upon reasonable request.
