# Supplementary material for: Botulinum toxin-induced masseter muscle atrophy is associated with impaired autophagic flux without signs of apoptosis in mice
Source: Cell Death Discov. 2026 Feb 28;12:121. doi: 10.1038/s41420-026-02982-7 (PMC13031692; doi:10.1038/s41420-026-02982-7)

Antibody	Brand	Country	Catalogue Number	Dilution Factor	Incubation	T°	Tech-nique
Caspase-3	Cell Signaling	MA, USA	#9662	1:1000	Overnight	4°C	WB
AIF	Cell Signaling	MA, USA	#4642	1:1000	Overnight	4°C	WB
Cleaved PARP	Cell Signaling	MA, USA	#5625	1:1000	Overnight	4°C	WB
LC3	Cell Signaling	MA, USA	#4108	1:1000<br>1:200	Overnight<br>Overnight	4°C<br>4°C	WB<br>IF
p62	Cell Signaling	MA, USA	#5114	1:1000	Overnight	4°C	WB
p62	Abnova	Taipei, Taiwan	#H00008878-M01	1:300	Overnight	4°C	IF
BAG3	Abcam	CB, UK	#ab47124	1:1000	Overnight	4°C	WB
GAPDH	Cell Signaling	MA, USA	#97166	1:1000	Overnight	4°C	WB
Anti-mouse	Cell Signaling	MA, USA	#7076	1:1000	1 h	RT	WB
Anti-rabbit	Cell Signaling	MA, USA	#7074	1:1000	1 h	RT	WB
Caveolin-3	BD Transduction Laboratories	NJ, USA	#610421	1:200	Overnight	4°C	IF
Anti-mouse Alexa Fluor 594	Invitrogen	ONT, Canada	#A21422	1:200	1 h	RT	IF
Anti-rabbit-Alexa Fluor 488	Invitrogen	ONT, Canada	#A21206	1:200	1 h	RT	IF

Table S1. Summary of antibodies used.
T°: Temperature; RT: Room temperature; WB = Western blot; IF= Immunofluorescence.

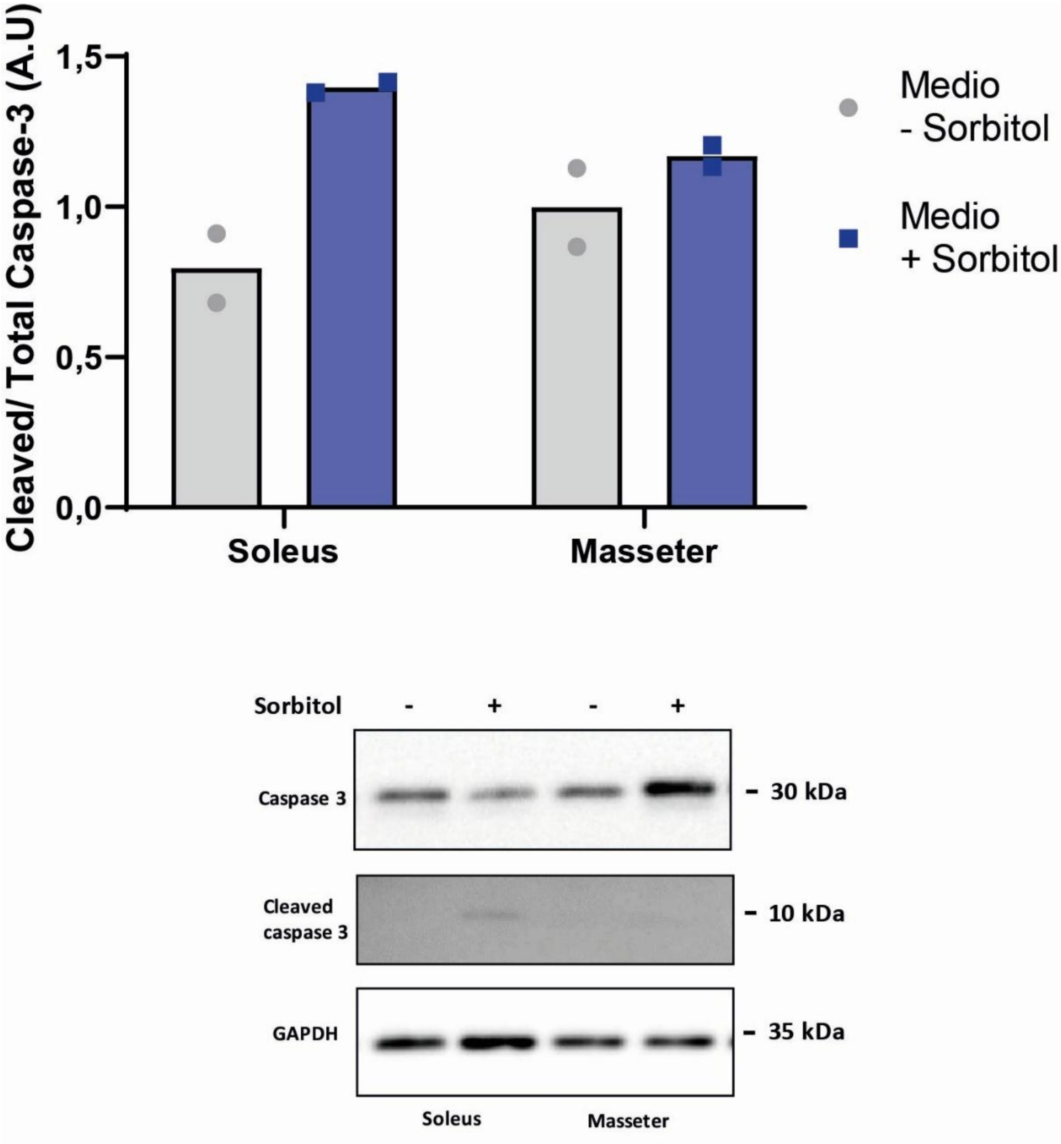

Figure S1. Osmotic stress increases cleaved caspase 3 in soleus muscle but not in the masseter muscle.

Dissected soleus and masseter muscles were incubated in vitro in a medium with 300 mM sorbitol for 12 hours to induce osmotic stress (n=2). Cleaved caspase-3 and total caspase-3 were evaluated by Western blotting. GAPDH was used as a loading control. A representative blot image and a graph showing the densitometric quantification of the bands are presented.

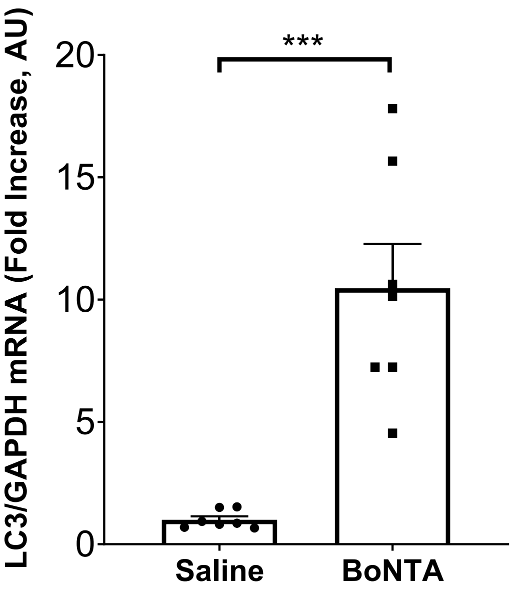

Figure S2. BoNTA increases LC3 mRNA levels in the masseter muscle.

Contralateral masseter muscles were injected with 0.2 U of BoNTA or saline. mRNA was extracted from muscles dissected 7 days after intervention and analyzed by RT-qPCR. GAPDH was used as housekeeping. BoNTA increased the mRNA levels at 7 days. Values are presented as mean ± SEM. (n = 7). * $p < 0.05$, ** $p < 0.01$, *** $p < 0.001$; Mann-Whitney test.

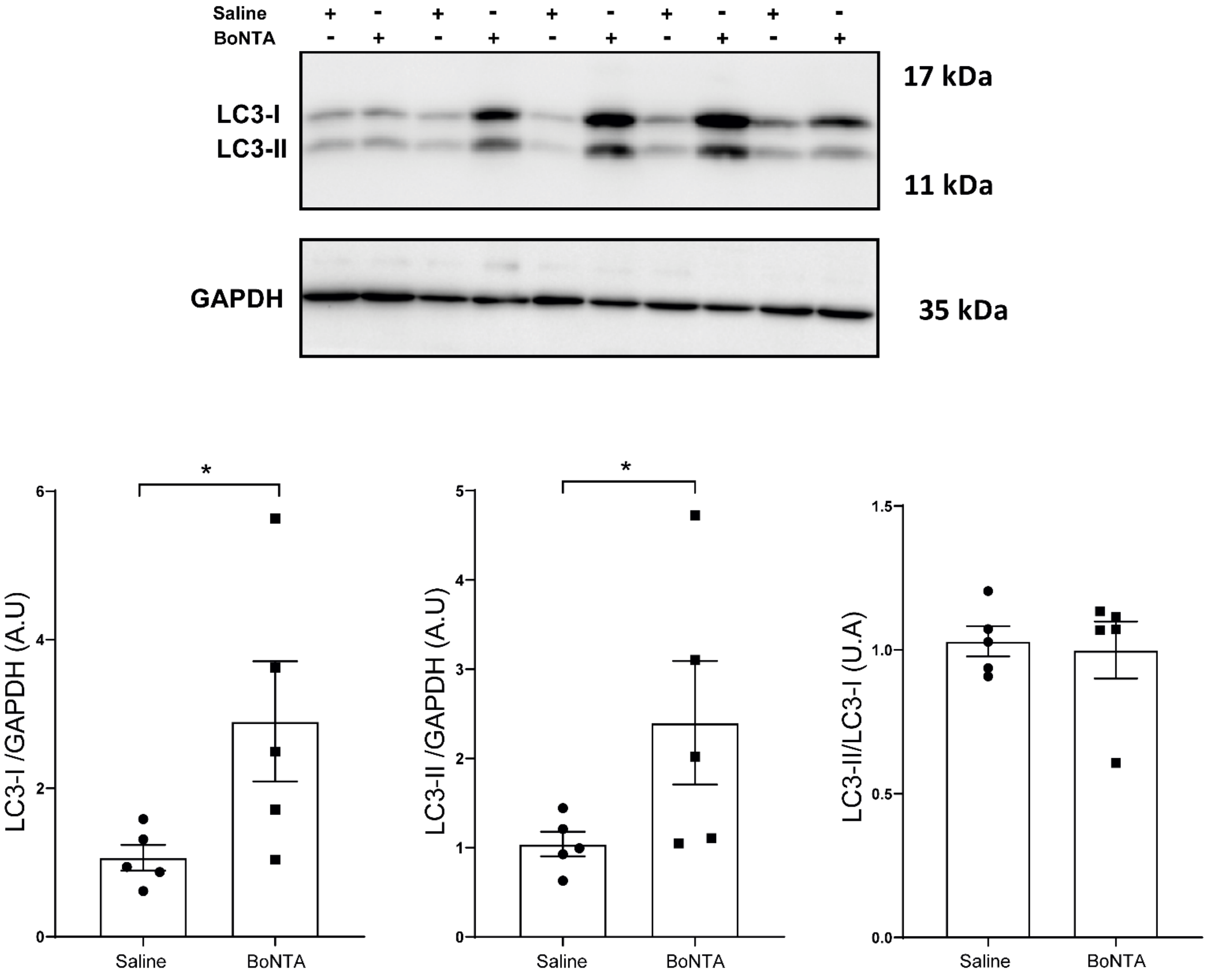

Figure S3. BoNTA increases LC3-I and LC3-II protein levels in the masseter muscle at 14 days.

Contralateral masseter muscles were injected with 0.2 U of BoNTA or saline solution. Proteins were extracted from muscles dissected 14 days after intervention and analyzed using Western blot. GAPDH was used as a normalizer. Representative blot image and corresponding densitometric quantification graphs are shown. Values are presented as mean ± SEM. (n = 5). * $p < 0.05$, ** $p < 0.01$, *** $p < 0.001$; Mann-Whitney test.

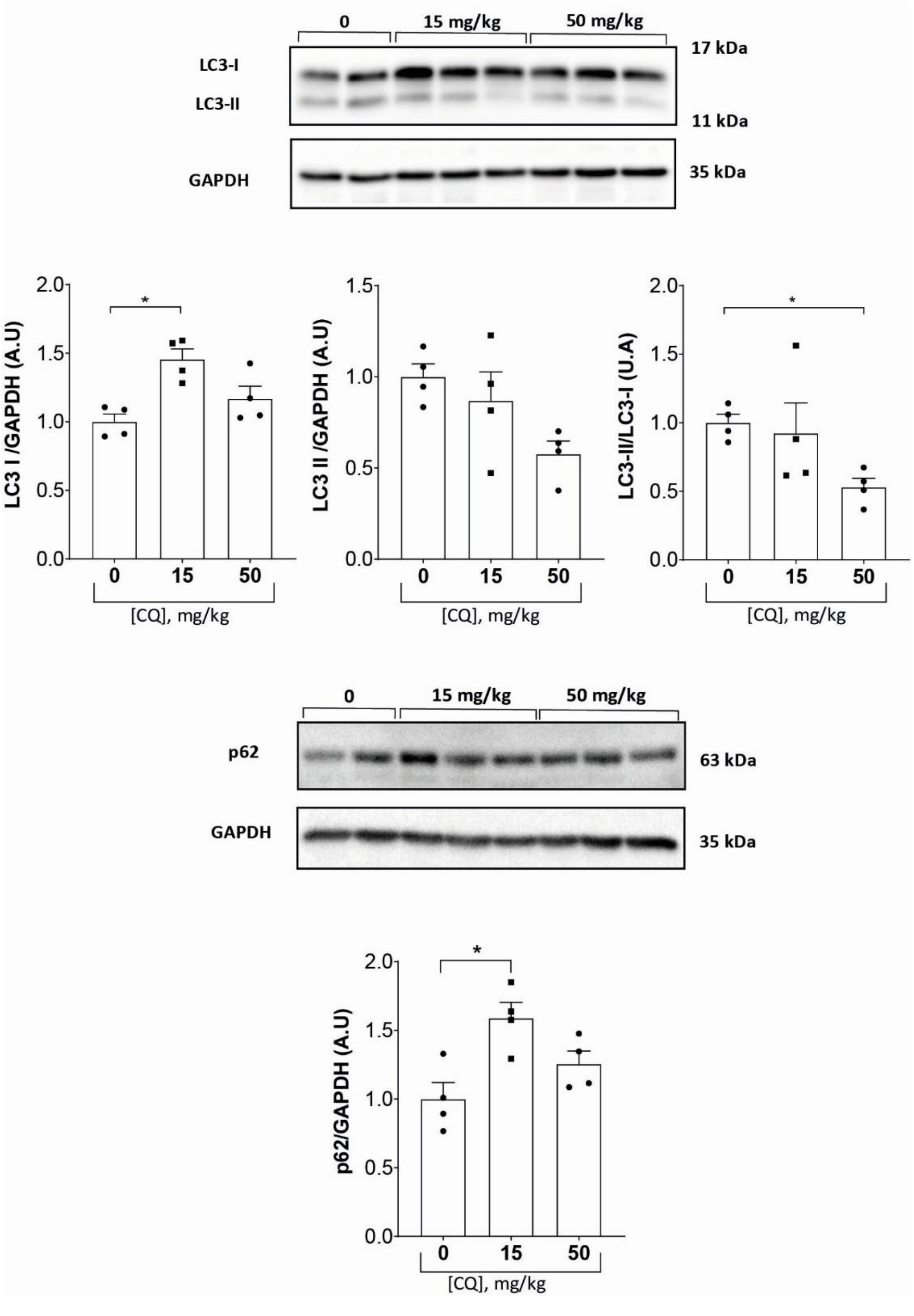

Figure S4. Chloroquine blocks autophagy in the masseter muscle in a dose-dependent manner.

Male BalbC mice were injected intraperitoneally (IP) with saline (0) or chloroquine (CQ) at doses of 15, 50, or 100 mg/kg. Injections were administered on days 0, 2, 5, and 7. On day 8, the animals were euthanized, and muscle samples were collected. The 100 mg/kg CQ dose was lethal, causing death in the treated mice. Proteins were extracted from the masseter muscles and analyzed using Western blot. GAPDH was used as a normalizer. A representative blot image and the corresponding densitometric quantification graphs are shown. 15 mg/kg and 50 mg/kg CQ decreased the LC3-II/LC3-I ratio in the masseter muscle (a), while only the 15 mg/kg dose increased p62 levels (b). Values are presented as mean ± SEM (n = 4). *$p < 0.05$. Kruskal-Wallis with Dunn´s post hoc.

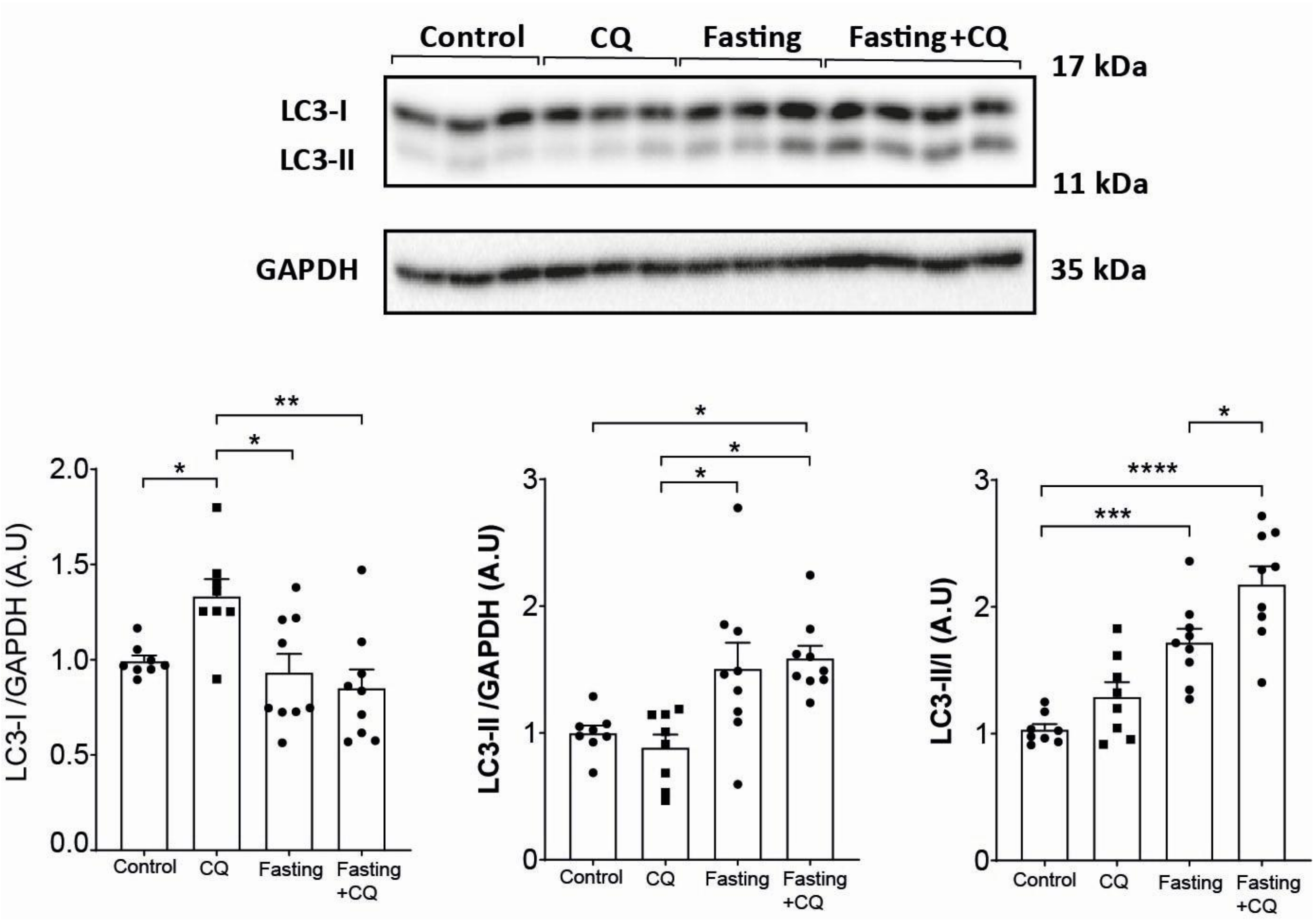

Figure S5. Fasting increases autophagy in the masseter muscle.

Adult male BalbC mice were injected IP with saline or 15 mg/kg chloroquine (CQ) on days 0, 2, 5 and 7. On day 7, animals were subjected to 16 hours of either a normal diet or dry fasting, during which food was withheld. On day 8, the animals were euthanized, and muscle samples were collected. Proteins were extracted from the masseter muscles and analyzed by Western blot. GAPDH was used as a normalizer. A representative blot image and the corresponding densitometric quantification graphs are presented. CQ treatment potentiated the increase in the LC3-II/LC3-I ratio induced by fasting. Values are presented as mean ± SEM (n = 8-9). *, $p < 0.05$; ***, $p < 0.001$. Kruskal-Wallis with Dunn´s post hoc.

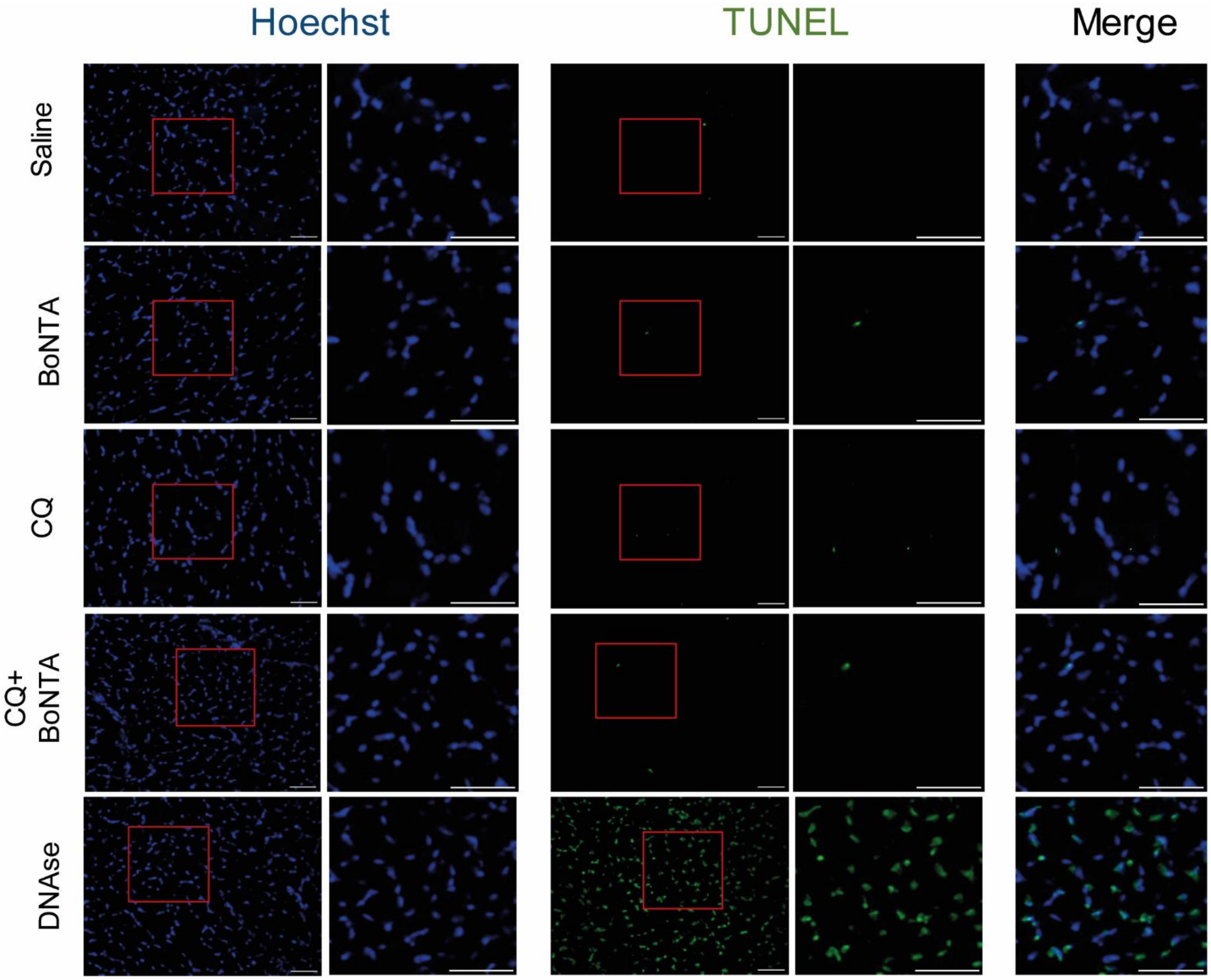

Figure S6. Injection of BoNTA together with chloroquine does not induce changes in DNA fragmentation in the masseter muscle.

Adult male BalbC mice were injected IP with saline or 15 mg/kg chloroquine (CQ) on days 0, 2, 5, and 7. On day 1, animals received an intramuscular injection of BoNTA into the right masseter and saline into the left masseter muscle. On day 8, the animals were euthanized, and samples were collected. The masseter muscles were dissected, frozen, and processed for transverse cryosections. TUNEL staining was evaluated as an indicator of fragmented DNA (green) and nuclei were labeled with Hoechst (blue). Representative images captured under an epifluorescence microscope are shown. Each panel includes a wide-field image, and an enlarged view of the area outlined in red. Cryosections treated with DNase served as positive controls for DNA fragmentation. In all images, the white scale bar represents 50 µm.

Figure S7. Uncropped original western blots. Panel **(A)** shows representative images of Caspase-3, AIF, and PARP after BoNTA injection shown in Manuscript Figure 1. Panel **(B)** displays representative images of LC3, p62, and BAG3 after BoNTA injection shown in Manuscript Figure 2. Panel **(C)** illustrates LC3 and p62 expression following intraperitoneal (IP) injection of CQ and BoNTA into the masseter muscle shown in Manuscript Figure 3. The black square indicates the section included in the original article.

A. Representative WB - Figure 1

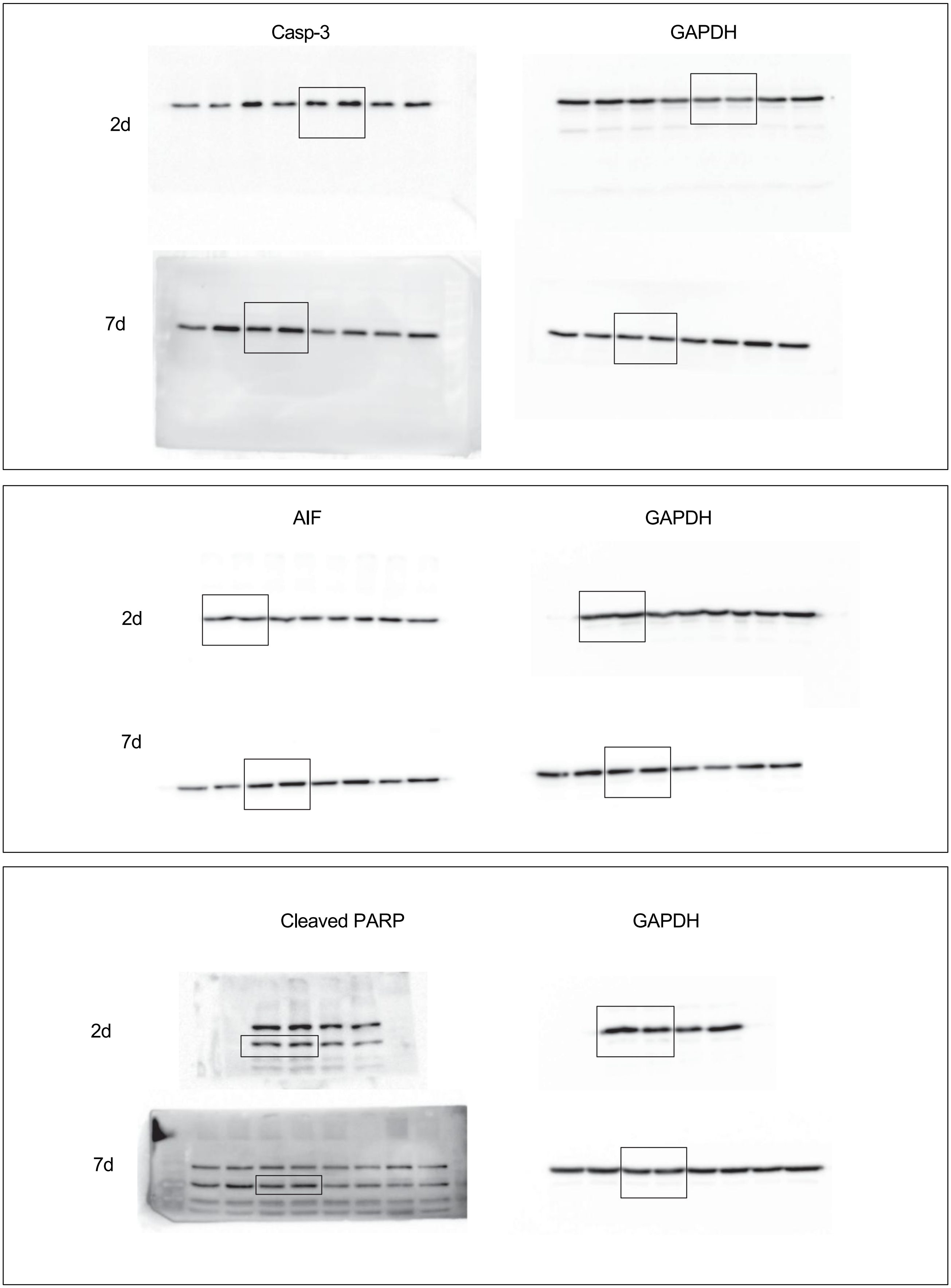

B. Representative WB - Figure 2

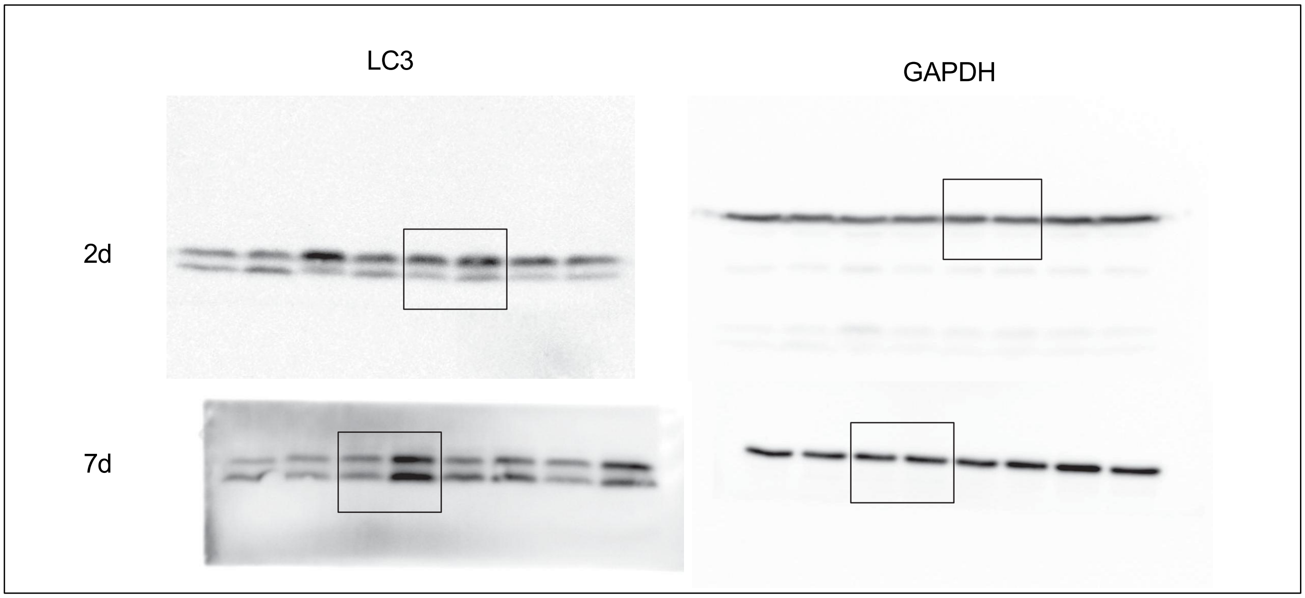

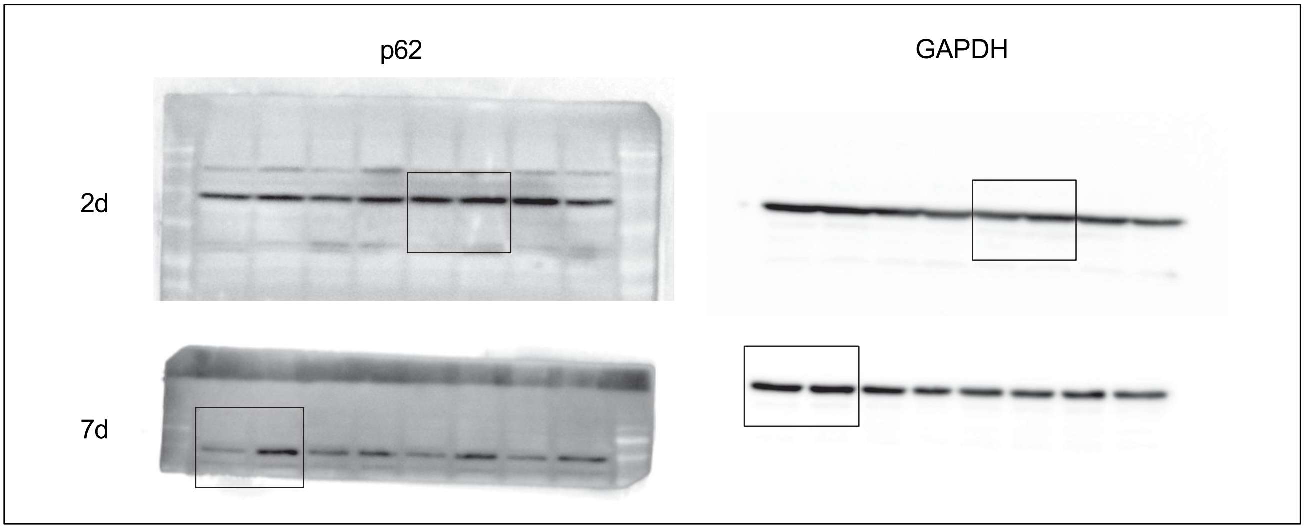

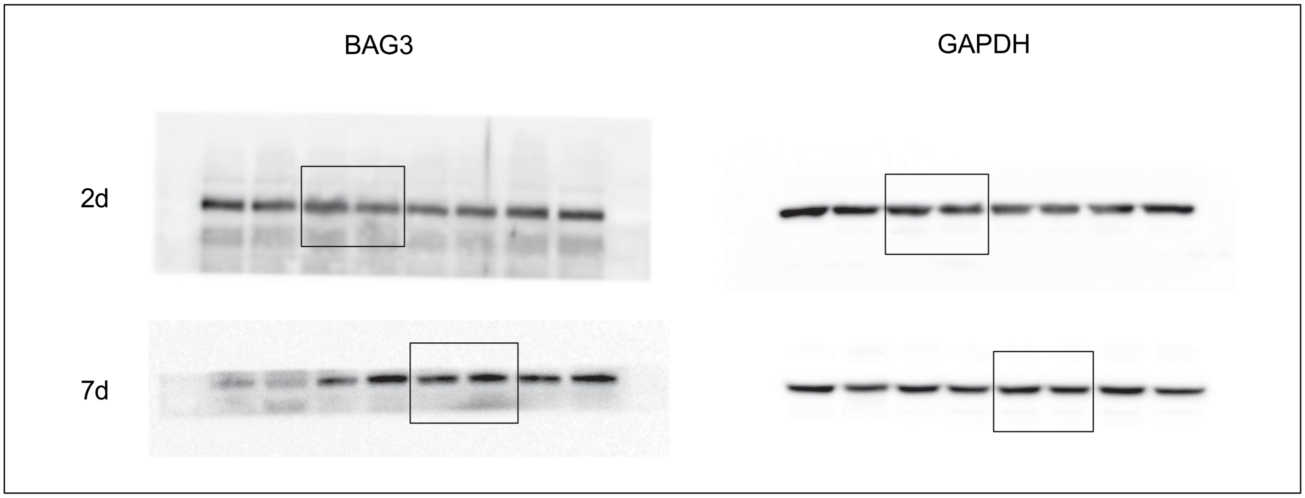

C. Representative WB - Figure 3

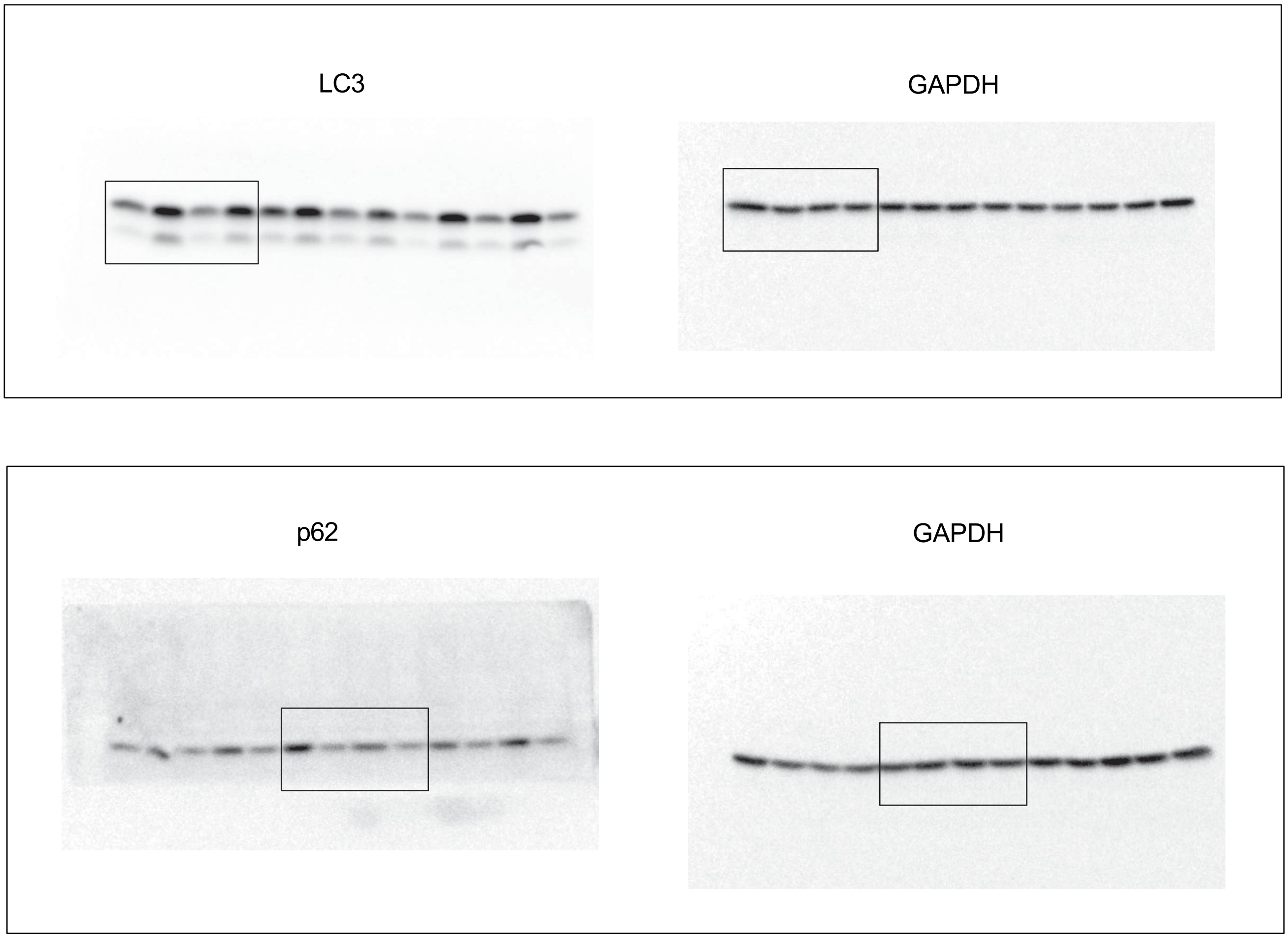

Supplement: Supplementary file 1 — Supplemental material [file 41420_2026_2982_MOESM1_ESM.pdf]
